# From Usher syndrome to Bardet-Biedl syndrome: Diagnosis after an atypical presentation 

**DOI:** 10.5414/CNCS111975

**Published:** 2026-03-31

**Authors:** Joaquim Milheiro, Raquel Pinto, Catarina Veiga, Adriana Dias, Cátia Pêgo, Sérgio Lemos

**Affiliations:** Nephrology Department, Local Health Unit Viseu Dão-Lafões, Viseu, Portugal

**Keywords:** ciliopathies, genetic testing, sensorineural hearing loss, retinitis pigmentosa

## Abstract

Syndromic associations of kidney and ophthalmological diseases are not typical in the young adult. The most common kidney manifestations are malformations of the genitourinary apparatus, while some syndromes can also present with kidney parenchymal disease. We report on a 51-year-old woman evaluated in the nephrology clinic for 12 months due to abnormal kidney function. She had been diagnosed with Usher syndrome due to the development of retinitis pigmentosa and sensorineural hearing loss in the last 20 years, without genetic testing. She had a family history of parental consanguinity, and disperse cardiac disease, congenital malformations, and congenital deafness. For chronic kidney disease of unknown etiology, a kidney biopsy was performed which revealed focal segmental glomerulosclerosis (FSGS) of the perihilar variant. This diagnosis prompted genetic testing that identified a mutation on gene SDCCAG8: c.397G>T, known for causing Bardet-Biedl type 16 and Senior-Løken type 7 syndromes. This case of perihilar FSGS is atypical in the setting of a ciliopathy and absence of metabolic or cardiovascular risk. Though urinary tract malformations and kidney disease can be expected, glomerular disease is not described in the literature. Genetic syndromic diagnoses require genetic screening due to the overlap of different symptoms and variable penetrance. The diagnosis of genetic diseases requires a high degree of suspicion, especially when the phenotype of the kidney disease is unusual. Identification of variants can help identify individuals who can benefit from genetic counseling.

## Introduction 

Syndromic presentation of kidney disease is a modern clinical challenge. With the development and growing availability of genetic testing, correct screening is fundamental in providing an adequate diagnosis, prevent identification of irrelevant and confounding mutations, and optimize spending in a still expensive and resource consuming field. Late-onset and slow-progression genetic phenotypes represent a challenge due to milder suspicion for known syndromes whose clinical variability might still be poorly understood. 

Bardet–Biedl syndrome (BBS) is an autosomal recessive disorder caused by loss-of-function mutations that can occur in at least 24 genes whose function is closely related to the primary cilium [1, 2, 3]. It is a multisystem non-motile ciliopathy characterized by developmental and multiorgan defects, most commonly retinal dystrophy, polydactyly, obesity, genital abnormalities, urinary anomalies, learning difficulties/cognitive impairment. Other manifestations might include speech delay, developmental delay, diabetes mellitus, dental anomalies, congenital heart disease, brachydactyly, ataxia, hyposmia. Other genetic ciliopathies associated with kidney disease are varied and can present with retinal degeneration, hearing defects, neurodevelopmental abnormalities, and even be as severe as to entail fetal and perinatal mortality. 

## Case presentation 

We report on a 51-year-old woman referred to the nephrology clinic for abnormal kidney function (creatinine of 1.4 mg/dL) that had been known for 12 months. She had been to ophthalmology and otorhinolaryngology clinic for vision loss for 20 years in the setting of retinitis pigmentosa (RP) and sensorineural hearing loss (SNHL) for 10 years. She had a presumed diagnosis of Usher syndrome, without genetic testing. She had also had a minor ischemic cerebral lesion diagnosed and a heterozygous MTHFR A1298C mutation detected. Family history revealed parental consanguinity, cardiac disease in her mother and maternal grandmother, 2 of 6 paternal uncles with congenital malformations, and 2 cousins with congenital SNHL. No other family member had been diagnosed with Usher syndrome or other genetic disease. 

Biochemical investigation confirmed chronic kidney disease (CKD) (estimated glomerular filtration rate of 42 mL/min/1.73m^2^) and found proteinuria of 500 mg per day with mild leukocyturia. The other biochemical, serological (including CMV, EBV, and parvovirus B19), and autoimmunity tests were unremarkable. Ultrasound of the kidney showed normal size with normal parenchymal to sinus differentiation. 

**Figure 1.**
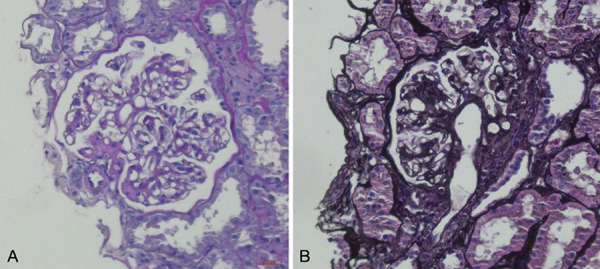
Kidney biopsy of focal segmental glomerulosclerosis (FSGS) lesion. Glomerulus with vascular pole sclerosis with hyalinosis extending to the arteriolar pole, perihilar lesion of FSGS (a, periodic acid Schiff; b, Jones methenamine silver stain, ×400).

Due to CKD of unknown etiology she underwent a kidney biopsy. A left kidney needle biopsy showed a total of 8 glomeruli, 2 in global sclerosis and the remaining hypertrophic, 1 of which with a perihilar segmental glomerulosclerosis lesion (Figure 1). Interstitial fibrosis and tubular atrophy accounted for ~ 10% of the sample and showed low-grade arteriolar hyalinosis. The direct immunofluorescence microscopy fragment had a single glomerulus showing glomerular IgM and C3 segmental deposits, and electron microscopy was not done. The pathological diagnosis of perihilar variant of focal segmental glomerulosclerosis (FSGS) motivated a literature review of FSGS associations with Usher syndrome, RP, and SNHL. According to the literature, most kidney abnormalities in multisystem non-motile ciliopathies require a polycystic phenotype, nephronophthisis, or urological abnormalities. 

Clinical criteria were insufficient for a diagnosis associated with ciliary dysfunction, so we proposed gene analysis that would include genes involved in multisystem ciliopathies. A next-generation sequencing (NGS) exome panel analysis was run with copy number variation analysis. This showed a mutation on gene SDCCAG8: c.397G>T. Mutations on gene SDCCAG8 are known causes for Bardet-Biedl type 16 and Senior-Løken type 7 syndromes and can be involved in nephronophthisis-related ciliopathies (NPHP-RC). This is a very rare mutation, described in the Genome Aggregation Database with a frequency of 0.0005%. She was diagnosed with Bardet-Biedl type 16 syndrome and, along with family members at risk, was referred to genetic counseling. 

## Discussion 

Our patient presents with an unusual diagnosis for syndromic kidney disease. Though perihilar FSGS lesions are mostly associated with cardiovascular and metabolic abnormalities [4, 5, 6], association with monogenic disease relating to podocyte-related genes has also been described [7]. BBS, as most ciliopathies, can present with endocrine and metabolic dysfunction. Regardless, our patient had a normal BMI and no body dysmorphic features that would have been consistent with the pattern of lesion. Our work-up included a larger panel of genes due to this unusual feature. Usher syndrome is also a non-motile ciliopathy and is characterized by syndromic RP associated with SNHL. Though clinically and genetically diverse, three subphenotypes have been proposed according to symptom severity and onset [8]. Type 1 (USH1), the most severe, presents with profound deafness at birth, vestibular dysfunction, and night blindness early in childhood. Type 2 (USH2) presents with moderate to severe hearing loss apparent at birth with no detectable vestibular dysfunction and a slower rate of RP progression. Type 3 (USH3) has a later onset, and affected individuals have normal hearing at birth. Vestibular dysfunction may develop over time. Diagnosis ofUsher syndrome also requires both clinical and genetic criteria. Kidney disease is not characteristic of this syndrome. 

Though the previous diagnosis of Usher syndrome had been practical from an ophtalmology and hearing point of view, as therapy would only be supportive care, it fitted the picture only until kidney disease was identified. 

BBS is a heterogeneous disorder, with some genes being implied in kidney tissue-specific proteins [9, 10]. The Bbsome, a protein complex with function of cell signaling and intraflagellar transport, and gene mutations in its coding genes seem to be the main cause of BBS. The complex’s close interaction with other nephronophthisis (NPHP)-associated proteins also help explain the phenotypic variation between these disorders and justify their challenge in clinical diagnosis. 

Senior-Løken phenotypes are associated with NPHP, which through tubulointerstitial nephritis present with urine concentration defects and early symptoms of polyuria and thirst. Both Senior-Løken syndrome and NPHP are associated with cystic kidney disease and RP but not usually with hearing loss. BBS has variable penetrance and clinical presentation and its diagnosis requires both clinical and genetic criteria. The current most consensual diagnosis criteria, as suggested by Forsythe and Beales [11], rely on the presence of at least 4 features, 4 major or 3 major plus 2 minor (Table 1). 

Systemic non-motile ciliopathies from genetic cause that occur with kidney disease include others like autosomal recessive NPHP-RC resulting in infantojuvenile kidney failure [12, 13], Senior-Løken syndrome, a NPHP phenotype associated with RP [14], Joubert syndrome, a ciliopathy associated with marked neurodevelopmental defects [15], and Meckel-Gruber syndrome, a severe multiorgan phenotype with high embryonic and postnatal mortality [16]. 

With ever-growing accessibility and affordability of genetic diagnosis, many diseases have shifted paradigm, allowing for counseling and prevention of disease in future generations. The better understanding of disease presentation in these syndromes, linked through close networks within the tissue-specific and non-specific interactomes is warranted for better disease modeling, for identifying candidates for genetic screening, and for opportunities of health gains. A mindful approach to syndromic and non-syndromic genetic causes of kidney disease are fundamental to develop a better understanding of the variability of disease presentation, more accurate and earlier diagnoses, and avoidance of incidental findings. Better disease modeling from diagnosis and pathway identification allow for pharmacological opportunities through improvement and development of new therapies [17, 18, 19]. Referral to genetic counseling can help patients better understand the disease and enables informed decisions. 

## Conclusion 

Ciliopathies are rare genetic disorders with variable penetrance and clinical variabilities. Their diagnosis is challenging and requires a high index of suspicion in patients with familial disease and other symptoms of ciliary disease. Perihilar FSGS lesions can occur in the setting of these genetic diseases and thus cannot rule them out. For more accurate diagnoses, syndromic presentation including hearing loss and familial occurrences, even when mild or unclear, warrant exclusion of genetic diseases. Growing identification of rare mutations can improve diagnosis for affected individuals whose phenotype can be misleading. Genetic analysis and case reporting can help elucidate non-classic kidney manifestation of ciliary disease. 

## Authors’ contributions 

J. M.: manuscript and figure preparation, data analysis, and interpretation. R. P.: manuscript preparation, data analysis, and interpretation. C. V.: manuscript preparation, data analysis, and interpretation. A. D.: manuscript preparation, data analysis, and interpretation. C. P.: original idea, manuscript preparation, patient follow up and work supervision. S. L.: manuscript preparation, patient follow up and work supervision. 

## Funding 

This work has not received any contribution, grant or scholarship. 

## Conflict of interest 

The authors declare that there are no competing interests regarding the publication of this article. 

**Table 1.** Clinical features for the diagnosis of Bardet-Biedl syndrome. For diagnosis, 4 major features or 3 major plus 2 minor are required. Adapted from Forsythe and Beales (2013) [11].

**Major feature**	**Minor feature**
Retinal cone-rod dystrophy	Neurologic abnormalities
Central obesity	Olfactory dysfunction
Postaxial polydactyly	Oral/dental abnormalities
Cognitive impairment	Cardiovascular and thoraco-abdominal abnormalities
Hypogonadism & genitourinary abnormalities	Gastrointestinal abnormalities
Kidney disease	Endocrine and metabolic abnormalities

## References

[1] Fliegauf M Benzing T Omran H When cilia go bad: cilia defects and ciliopathies. Nat Rev Mol Cell Biol. 2007; 8: 880–893. 17955020 10.1038/nrm2278

[2] Reiter JF Leroux MR Genes and molecular pathways underpinning ciliopathies. Nat Rev Mol Cell Biol. 2017; 18: 533–547. 28698599 10.1038/nrm.2017.60 PMC5851292

[3] Mitchison HM Valente EM Motile and non-motile cilia in human pathology: from function to phenotypes. J Pathol. 2017; 241: 294–309. 27859258 10.1002/path.4843

[4] Fogo AB Causes and pathogenesis of focal segmental glomerulosclerosis. Nat Rev Nephrol. 2015; 11: 76–87. 25447132 10.1038/nrneph.2014.216 PMC4772430

[5] Darouich S Goucha R Jaafoura MH Zekri S Ben Maiz H Kheder A Clinicopathological characteristics of obesity-associated focal segmental glomerulosclerosis. Ultrastruct Pathol. 2011; 35: 176–182. 21657818 10.3109/01913123.2011.584657

[6] Stokes MB D’Agati VD Morphologic variants of focal segmental glomerulosclerosis and their significance. Adv Chronic Kidney Dis. 2014; 21: 400–407. 25168828 10.1053/j.ackd.2014.02.010

[7] Nagano C Hara S Yoshikawa N Takeda A Gotoh Y Hamada R Matsuoka K Yamamoto M Fujinaga S Sakuraya K Kamei K Hamasaki Y Oguchi H Araki Y Ogawa Y Okamoto T Ito S Tanaka S Kaito H Aoto Y Clinical, pathological, and genetic characteristics in patients with focal segmental glomerulosclerosis. Kidney360. 2022; 3: 1384–1393. 36176665 10.34067/KID.0000812022 PMC9416844

[8] Sorusch N Wunderlich K Bauss K Nagel-Wolfrum K Wolfrum U Usher syndrome protein network functions in the retina and their relation to other retinal ciliopathies. Adv Exp Med Biol. 2014; 801: 527–533. 24664740 10.1007/978-1-4614-3209-8_67

[9] M’hamdi O Ouertani I Chaabouni-Bouhamed H Update on the genetics of bardet-biedl syndrome. Mol Syndromol. 2014; 5: 51–56. 24715851 10.1159/000357054 PMC3977223

[10] Priya S Nampoothiri S Sen P Sripriya S Bardet-Biedl syndrome: Genetics, molecular pathophysiology, and disease management. Indian J Ophthalmol. 2016; 64: 620–627. 27853007 10.4103/0301-4738.194328 PMC5151149

[11] Forsythe E Beales PL Bardet-Biedl syndrome. Eur J Hum Genet. 2013; 21: 8–13. 22713813 10.1038/ejhg.2012.115 PMC3522196

[12] Hildebrandt F Attanasio M Otto E Nephronophthisis: disease mechanisms of a ciliopathy. J Am Soc Nephrol. 2009; 20: 23–35. 19118152 10.1681/ASN.2008050456 PMC2807379

[13] Luo F Tao YH Nephronophthisis: A review of genotype-phenotype correlation. Nephrology (Carlton). 2018; 23: 904–911. 29717526 10.1111/nep.13393 PMC6175366

[14] Tsang SH Aycinena ARP Sharma T Ciliopathy: Senior-Løken Syndrome. Adv Exp Med Biol. 2018; 1085: 175–178. 30578507 10.1007/978-3-319-95046-4_34

[15] Bachmann-Gagescu R Dempsey JC Phelps IG Disorders with extreme genetic heterogeneity. J Med Genet. 2015; 52: 514–522. 26092869 10.1136/jmedgenet-2015-103087 PMC5082428

[16] Hartill V Szymanska K Sharif SM Wheway G Johnson CA Meckel-Gruber Syndrome: an update on diagnosis, clinical management, and research advances. Front Pediatr. 2017; 5: 244. 29209597 10.3389/fped.2017.00244 PMC5701918

[17] Franceschini N Frick A Kopp JB Genetic testing in clinical settings. Am J Kidney Dis. 2018; 72: 569–581. 29655499 10.1053/j.ajkd.2018.02.351 PMC6153053

[18] Kaye C Korf B Genetic literacy and competency. Pediatrics. 2013; 132: S224–S230. 24298131 10.1542/peds.2013-1032G

[19] Patch C Middleton A Genetic counselling in the era of genomic medicine. Br Med Bull. 2018; 126: 27–36. 29617718 10.1093/bmb/ldy008 PMC5998955

